# Selective inhibition of Ebola entry with selective estrogen receptor modulators by disrupting the endolysosomal calcium

**DOI:** 10.1038/srep41226

**Published:** 2017-01-24

**Authors:** Hanlu Fan, Xiaohong Du, Jingyuan Zhang, Han Zheng, Xiaohui Lu, Qihui Wu, Haifeng Li, Han Wang, Yi Shi, George Gao, Zhuan Zhou, Dun-Xian Tan, Xiangdong Li

**Affiliations:** 1 State Key Laboratory of AgroBiotechnology, Faculty of Biological Sciences, China Agricultural University, Beijing, 100193, China; 2 State Key Laboratory of Biomembrane and Membrane Biotechnology and Beijing Key Laboratory of Cardiometabolic Molecular Medicine, Institute of Molecular Medicine and PKU-IDG/McGovern Institute for Brain Research and Peking-Tsinghua Center for Life Sciences, Peking University, Beijing 100871, China; 3 CAS Key Laboratory of Pathogenic Microbiology and Immunology, Institute of Microbiology, Chinese Academy of Sciences, Beijing 100101, China; 4 The University of Texas Health Science Center at San Antonio Department of Cellular and Structural Biology, San Antonio, TX 78229-3900, USA

## Abstract

The Ebola crisis occurred in West-Africa highlights the urgency for its clinical treatments. Currently, no Food and Drug Administration (FDA)-approved therapeutics are available. Several FDA-approved drugs, including selective estrogen receptor modulators (SERMs), possess selective anti-Ebola activities. However, the inhibitory mechanisms of these drugs remain elusive. By analyzing the structures of SERMs and their incidental biological activity (cholesterol accumulation), we hypothesized that this incidental biological activity induced by SERMs could be a plausible mechanism as to their inhibitory effects on Ebola infection. Herein, we demonstrated that the same dosages of SERMs which induced cholesterol accumulation also inhibited Ebola infection. SERMs reduced the cellular sphingosine and subsequently caused endolysosomal calcium accumulation, which in turn led to blocking the Ebola entry. Our study clarified the specific anti-Ebola mechanism of SERMs, even the cationic amphiphilic drugs (CADs), this mechanism led to the endolysosomal calcium as a critical target for development of anti-Ebola drugs.

Ebola virus, a member of the *Filoviridae* family, is an enveloped RNA virus that causes hemorrhagic fever in humans and non-human primates with species dependent lethality ranging from 50 to 90%^1^,^2^. Entry of Ebola virus into the cells, which is mainly mediated by its sole glycoprotein (GP), is a target for therapeutic intervention^3^,^4^,^5^,^6^,^7^. Ebola entry is unusual in that it requires proteolytic-priming of GP followed by engagement of Niemann-Pick C1 (NPC1)^8^,^9^,^10^,^11^, Recently, it has been proven that the Ebola virus enters cells through the endolysosome that contain both NPC1 and the two-pore segment channel 2 (TPC2)^12^.

As of March 31, 2016, the recent Ebola crisis in West Africa (Guinea, Sierra Leone, and Libya) reportedly cause 11,323 deaths^13^. Unfortunately, there are still no FDA-approved vaccines or anti-Ebola therapeutics^1^,^14^. Considering the time-consuming process of developing new drugs, it is more cost- and time-effective to screen for effective anti-Ebola drugs from among the drugs that are already FDA-approved. Some groups have identified 80 FDA-approved drugs with selective anti-Ebola activity, including the SERMs^3^,^4^,^6^,^7^. Many of these identified drugs selectively inhibit the infection of Ebola virus and do not inhibit vesicular stomatitis virus (VSV) or the lymphocytic choriomeningitis virus. While the mechanism of inhibition is unknown, it is clear that these drugs do not inhibit the internalization of Ebola virus to the lysosomal associated membrane protein 1 (LAMP1)-positive lysosome, the acidification of the endolysosome, or the processing of Ebola GP cathepsin^3^,^4^,^6^. Moreover, the inhibition of Ebola entry by SERMs is independent of expression of the estrogen receptors, making the mechanism by which SERMs inhibit Ebola infection difficult to ascertain^15^.

Recently, it is reported that toremifene can bind to and destabilize the Ebola GP trimer, and trigger premature release of GP2, thereby preventing fusion between the viral and endolysosomal membranes^16^. Compared to the ethyl chlorine of toremifene, the corresponding groups in tamoxifen, clomiphene and raloxifene make weaker (even no) interations with the binding sites in Ebola GP^16^ (Supplementary Fig. S1). However, tamoxifen, clomiphene and raloxifene all can effectively inhibit the Ebola infection^3^,^4^,^6^,^7^, making the interaction with Ebola GP not the key mechanism in Ebola inhibition by these SERMs. These uncertain mechanisms need further studies.

Interestingly, 45 of the 80 drugs found to have selective anti-Ebola activity are CADs, including the SERMs. Moreover, several studies have showed that some of the CADs (including the SERMs) have some incidental biological activities, *i.e.,* drug-induced phospholipidosis (DIP)^17^,^18^,^19^,^20^,^21^, cholesterol accumulation^22^, steatohepatitis^23^,^24^,^25^,^26^, and functional inhibition of acid sphingomyelinase (ASM) and acid ceramidase (AC)^27^,^28^. Much of the incidental biological activities of the CADs are related to their structural properties; several CADs are weak bases which are protonated after entering the low pH of the endolysosome. The ionized form of the CADs cannot pass through the hydrophobic core of the phospholipid bilayer that surrounds the endolysosome compartment, and are, therefore, trapped and concentrated inside^17^. The concentrated CADs in the endolysosome could disturb phospholipid metabolism, the transport of lipids and proteins, and the activity of enzymes in the endolysosome.

Based on the above facts, we hypothesized that these incidental biological activities of the CADs could be a plausible mechanism for the observed inhibition of Ebola infection. In this study, we explored the relationships between Ebola infection inhibition and the adverse effect of SERMs. Using an LC-MS/MS assay and imaging of endolysosomal calcium release, we demonstrated that SERMs could reduce the cellular sphingosine levels and subsequently induce accumulation of endolysosomal calcium. Moreover, chelating luminal endocytic calcium with high-affinity Rhod-dextran induced the specific inhibition of Ebola infection without inhibiting the internalization of Ebola virus-like particle (VLP) to the TPC2+ endolysosome. These data support our hypothesis that SERMs (and potentially other CADs) induce sphingosine defects and subsequent endolysosomal calcium accumulation, which in turn inhibit the release of Ebola from the TPC2+ endolysosome.

## Results

### SERMs selectively inhibit the entry of the Ebola pseudovirion, but not through the estrogen receptor pathway

**Figure 1:**
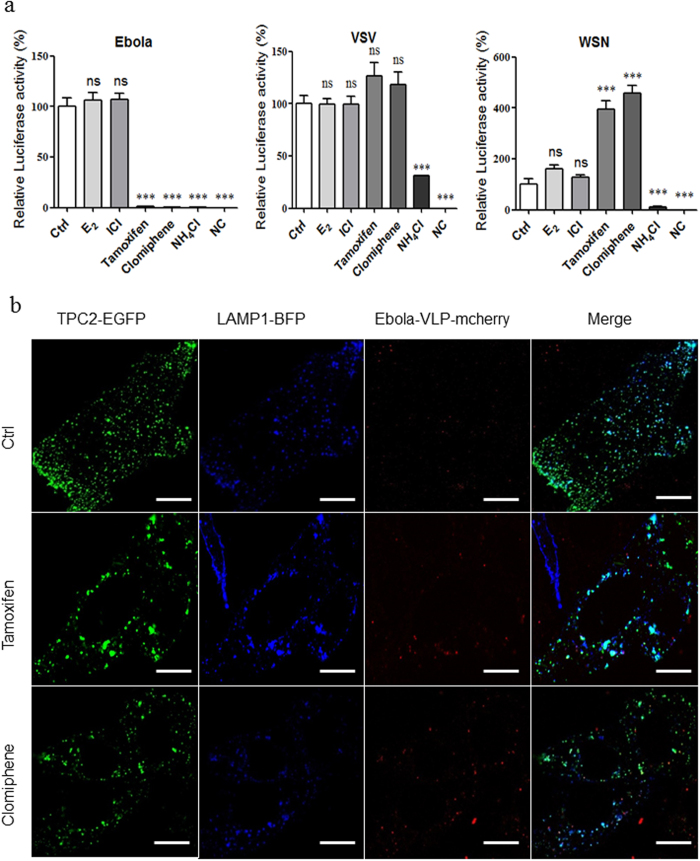
SERMs selectively inhibit the entry of Ebola pseudovirion, but not through the estrogen receptor pathway. (**a**) Effects of E_2_, ICI, tamoxifen, and clomiphene on Ebola/WSN/VSV pseudovirion entry. HepG2 cells were pretreated with 10 nM E2, 100 nM ICI, 10 μM tamoxifen, and 10 μM clomiphene for 1 h and infected by Ebola/WSN/VSV pseudovirion with compounds for 24 h. The cells were then lysed, and the luciferase assay was carried out. NH_4_Cl serves as a negative control. (**b**) Representative images of colocalization of VLPs (red, marked by mCheery-VP40) with TPC2 (green, marked with EGFP) and LAMP1 (blue, marked with BFP) from 10 μM tamoxifen or 10 μM clomiphene treated HepG2. White arrows indicate examples of colocalization. Data are expressed as the means ± SEM (n = 3). Significant differences versus control group are presented by asterisks (*), **P < 0.01, ***P < 0.001, ns means no significance.

As all stages of Ebola entry (binding to and internalization from the cell surface, as well as trafficking to and fusion with the limiting membrane of endolysosome) are mediated by trimeric GP spikes arrayed around the Ebola particles^29^,^30^, so the pseudovirion model with Ebola GP can simulate the stages of wild-type Ebola entry, and several groups have studied the Ebola entry mechanism and screened the anti-Ebola entry drugs with Ebola pseudovirion model^3^,^6^,^31^,^32^. We choose the pNL4-3-Luc-R^−^E^−^ based Ebola pseudovirion to study the Ebola entry, and the Ebola VLP-mcherry (composed by GP, VP40 and VP40-mcherry) to study the Ebola internalization. Then, we treated the hepatocellular carcinoma cell line HepG2 with two SERMs (tamoxifen and clomiphene), and infected them with the Ebola pseudovirion. Both SERMs selectively inhibited the Ebola entry more than 90%; however, they did not inhibit the entry of the VSV or Influenza A (A/WSN/33(H1N1), WSN) pseudovirions (Fig. 1a). Treatment with estrogen and estrogen receptor (ER) antagonists did not inhibit the Ebola pseudovirion entry (Fig. 1a), excluding the possibility that SERMs inhibit the Ebola entry through the ER pathway.

To determine the effect of SERMs on Ebola internalization, we transfected the HepG2 cells with the TPC2-EGFP and LAMP1-BFP vectors, and infected the cells with Ebola VLP-mCherry. Co-localization of TPC2-EGFP, LAMP1-BFP and VLP-mCherry demonstrated that SERMs did not inhibit Ebola internalization to the TPC2+ lysosome; rather, they blocked Ebola VLP release from the lysosome (Fig. 1b).

### SERMs do not interact with NPC1 domain A or domain C

It was reported that U18666a, also a CAD, interacts with and inhibits NPC1, and also inhibits the Ebola entry^8^,^33^. We also observed that U18666a selectively inhibited the entry of Ebola pseudovirion (no entry inhibition was observed for the VSV or WSN pseudovirions) and did not inhibited Ebola VLP internalization to the TPC2+ lysosome (Supplementary Fig. S2a, S2b). Considering the critical role of NPC1 domain C in Ebola entry in our previous study and the similar structural properties of the CADs displaying selective anti-Ebola activity^34^, we speculated that the CADs with selective anti-Ebola activity may target NPC1 to inhibit Ebola entry. To test this possibility, we expressed and purified the domains A and C of NPC1^34^,^35^. Then, we carried out MicroScale Thermophoresis (MST) and Isothermal Titration Calorimetry (ITC) to detect the interactions between tamoxifen, clomiphene, or U18666a and the NPC1 domains A and C. In all cases, we did not detect interactions by MST or ITC (Supplementary Fig. S3a, S3b, respectively).

### Equal dosages of SERMs inhibit Ebola pseudovirion entry and induce cholesterol accumulation

**Figure 2:**
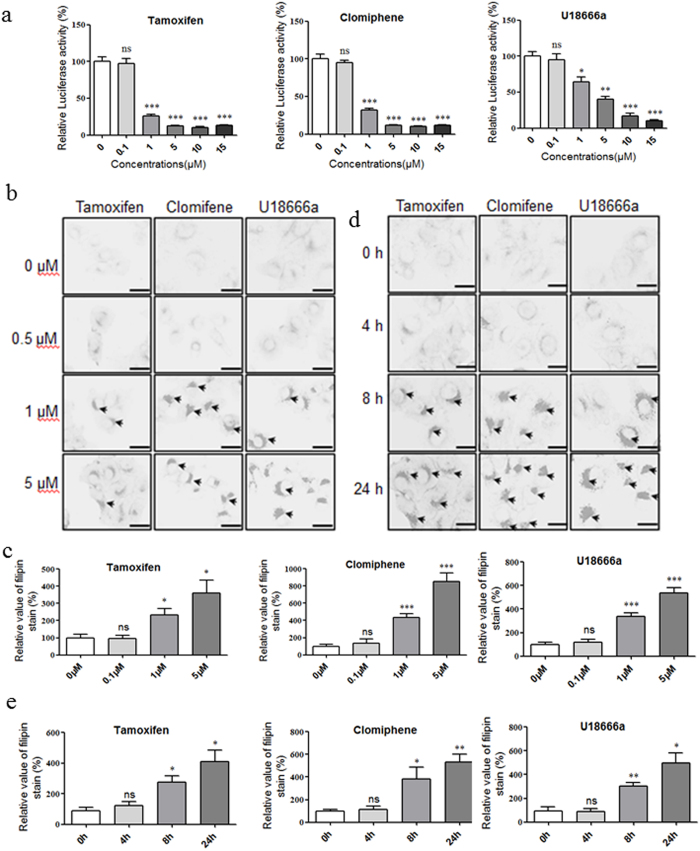
Equal dosages of SERMs inhibit Ebola pseudovirion entry and induce cholesterol accumulation. (**a**) Dose-response inhibition of Ebola entry for tamoxifen, clomiphene and U18666a. Data are expressed as the means ± SEM (n = 3). (**b**) Dose-response cholesterol accumulation for tamoxifen, clomiphene and U18666a. (**b**) Time-response cholesterol accumulation for tamoxifen, clomiphene and U18666a. Black arrows indicate examples of cholesterol accumulation. (**d,e**) The quantification of cholesterol accumulation stained with filipin. Data are expressed as the means ± SEM (n ≥ 4). Significant differences versus control group (0 μM or 0 h) are presented by asterisks (*), *P < 0.05, **P < 0.01, ***P < 0.001, ns means no significant.

Considering the similar structural properties and the reported incidental biological activities of the CADs, it is possible that the incidental biological activities of the SERMs are related to the mechanism of Ebola entry inhibition by the SERMs. To verify this hypothesis, we compared the effective SERMs dosages for both cholesterol accumulation and Ebola entry inhibition. The Ebola pseudovirion assay demonstrated that SERMs inhibit Ebola entry starting from 1 μM (Fig. 2a). Filipin staining also indicated that SERMs induce cholesterol accumulation at approximately 1 μM (Fig. 2b,d, Supplementary Fig. S4). Therefore, the dosages of SERMs required to induce cholesterol accumulation matches the dosages of SERMs required for Ebola entry inhibition, which implies that this incidental biological activity of SERMs are related to the Ebola entry inhibition mechanism. Intriguingly, the cholesterol accumulation appeared after 8 h of SERMs incubation (Fig. 2c,e), whereas the Ebola virus has already entered the cytoplasm at approximately 2 h^36^. Thus, we speculated that intercellular changes within 2 h after SERM treatment should mediate the cholesterol accumulation and the inhibition of Ebola entry.

### SERMs reduce the cellular sphingosine

**Figure 3:**
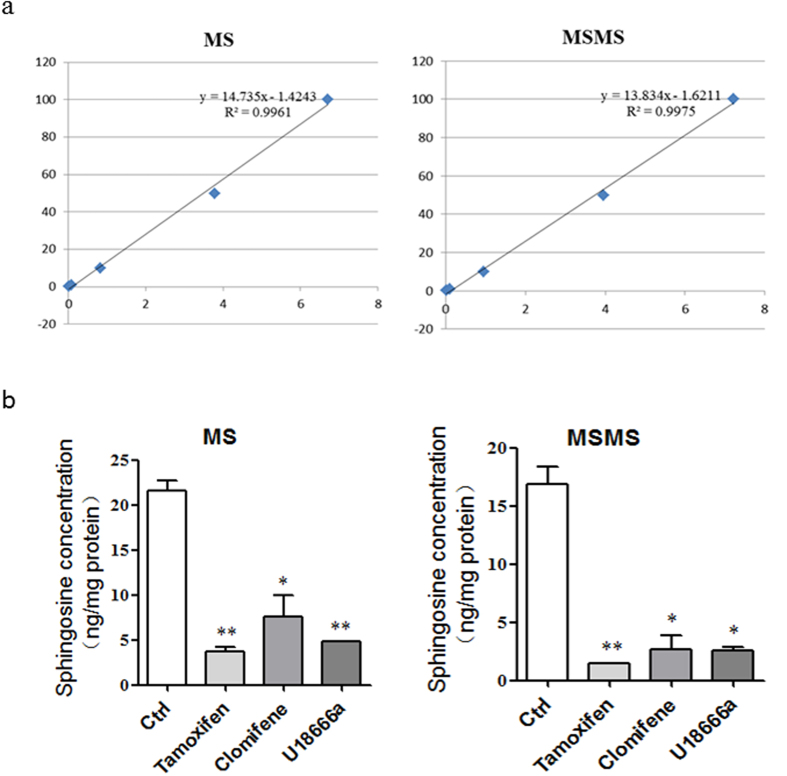
SERMs reduce the cellular sphingosine. (**a**) Standard curves for quantification of Sph (0.1–100 ng/mL) with C17-Sph (10 ng/mL) as the internal standard. (**b)** Measurement of changes in intracellular levels of Sph in HepG2 cells. Cells were treated with 10 μM tamoxifen, clomiphene or U18666a for 1 h, then lipids were extracted and the levels of Sph were analyzed. Data are expressed as the means ± SEM (n = 3). Significant differences versus control group are presented by asterisks (*), ***P < 0.001.

As an Ebola entry inhibitor, U18666a induces sphingosine accumulation within 10 min; moreover, sphingosine treatment also induces cholesterol accumulation^37^. We speculated that SERMs may act in the same way as U18666a by inducing accumulation of sphingosine and cholesterol. To detect the effect of SERMs on sphingosine storage, we carried out the liquid chromatography–tandem mass spectrometry (LC-MS/MS). Surprisingly, both U18666a and SERMs significantly decreased the sphingosine of HepG2 and Hela cell lines after 1 h treatment (Fig. 3, Supplementary Fig. S5).

### SERMs upregulate the endolysosomal calcium levels

**Figure 4:**
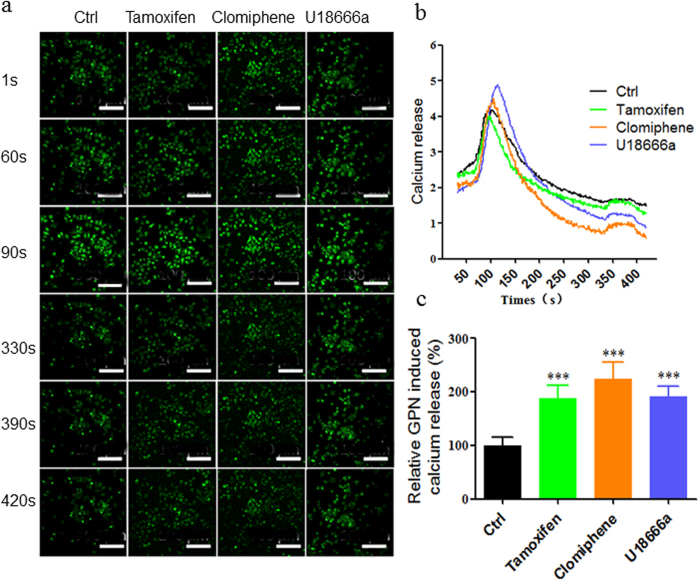
SERMs upregulate the endolysosomal calcium levels. (**a**) Representative images of the endolysosomal calcium release of HepG2 detected by Fluo8-AM calcium indicator at 1 s, 60 s, 90 s, 330 s, 390 s, and 420 s after incubation of 10 μM tamoxifen, clomiphene or U18666a for 1 h. (**b**) The calcium release of HepG2 loaded with 1 μM TG at 60 s for 30 s and 200 μM GPN at 330 s for 60 s. HepG2 cells were pre-treated with 10 μM tamoxifen, clomiphene or U18666a for 1 h. (**c**) The quantification of calcium release induced by 200 μM GPN. Data are expressed as the means ± SEM (n > 20). Significant differences versus control group are presented by asterisks (*), ***P < 0.001.

It has been reported that the sphingosine could regulate the levels of endolysosomal calcium^37^, and we demonstrated that SERMs could decrease sphingosine in this study. To establish a correlation between SERMs and endolysosomal calcium, we imaged Gly-Phe β-naphthylamide (GPN)-sensitive (endolysosomal) calcium release to detect the effect of SERMs on endolysosomal calcium levels. The results demonstrated that both SERMs and U18666a significantly up-regulated the calcium release from the GPN-sensitive endolysosomal calcium store after 1 h treatment (Fig. 4a–c).

**Figure 5:**
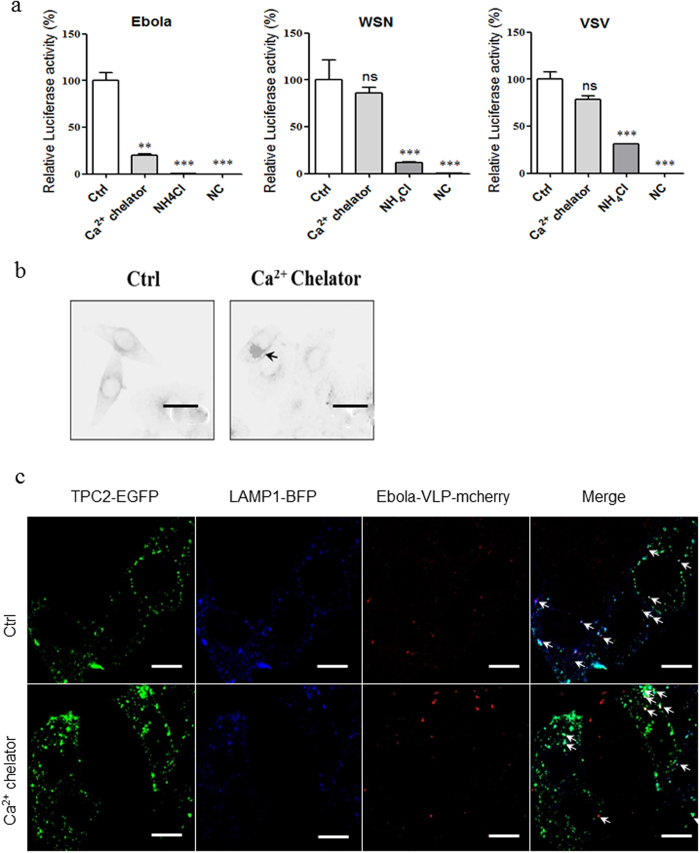
Chelation of endolysosomal calcium specifically inhibits the entry of Ebola pseudovirion. (**a**) Effects of endolysosomal calcium specific chelator on Ebola/WSN/VSV pseudovirions entry. HepG2 cells were pretreated with used 0.25 mg/ml calcium high-affinity Rhod-dextran for 1 h, and infected by Ebola/WSN/VSV pseudovirion with compounds for 24 h, then was lysed and to carry out the luciferase assay. NH_4_Cl serves as a negative control. (**b**) Representative images of co-location of VLPs (red, marked by mCheery-VP40) with TPC2 (green, marked with EGFP) and LAMP1 (blue, marked with BFP) from 0.25 mg/ml calcium high-affinity Rhod-dextran treated HepG2. White arrows indicate examples of colocalization. (**c**) Representative images of cholesterol accumulation in HepG2 induced by treatment of 0.25 mg/ml calcium high-affinity Rhod-dextran for 24 h. Black arrows indicate examples of cholesterol accumulation. Data are expressed as the means ± SEM (n = 3). Significant differences versus control group are presented by asterisks (*), **P < 0.01, ns means no significant.

To determine whether endolysosomal calcium is involved in the mechanism of Ebola entry inhibition, we treated HepG2 cell line with a high-affinity Rhod-dextran to chelate intraluminal calcium, and infected the cells with Ebola, WSN, or VSV pseudovirions. Interestingly, chelation of endolysosomal calcium significantly and specifically inhibited the entry of Ebola, while no inhibitory effects were observed for the VSV or WSN pseudovirions (Fig. 5a). Chelation did not inhibit the internalization of Ebola VLP to the TPC2+ lysosome (Fig. 5c). Moreover, chelation of endolysosomal calcium also induced the cholesterol accumulation (Fig. 5b).

## Discussion

SERMs were identified as new and specific inhibitors for Ebola infection from a screen of FDA approved drugs^3^,^4^,^6^,^7^; however, the definite mechanism by which the SERMs inhibit the infection of Ebola is still uncertain. Because the possibilities of the disturbed acidification, cathepsin activity, or internalization of Ebola have been excluded previously^4^, we attempted to ascertain the relationship between the inhibition of Ebola infection and the adverse effect of SERMs. In this study, our results indicated that both tamoxifen and clomiphene specifically inhibit the release of Ebola from TPC2+ lysosome, and this inhibition is independent of the estrogen receptor pathway.

Entry of Ebola virus into the cells, which is mainly mediated by its sole glycoprotein (GP)^29^,^30^, is initiated by the binding of Ebola GP to C-type lectins and the viral envelope phosphatidylserine to phosphatidylserine receptors^38^. Ebola virion is thought to be internalized into endolysosome primarily through micropinocytosis^39^,^40^,^41^, meanwhile the heavily glycosylated mucin-like domain and glycan cap of GP1 is removed by the low pH-dependent Cathepsins L and B, resulting in a 17- to 19-kDa GP1^42^,^43^. Proteolytic processing of GP expose the GP1 receptor binding domain (RBD), which interact with the NPC1 domain C within the endolysosome^44^,^45^. This interaction between GP1 and NPC1 domain C and low pH conditions are essential for subsequent membrane fusion^15^,^34^,^46^. However, whether there is other membrane fusion trigger factor remains unclear. Recently, it has been proven that TPC2 plays a key role in Ebola virus infection and the Ebola virus enters cells through the endolysosome that contain both NPC1 and TPC2^47^,^48^. Although the definite role of TPC2 is uncertain, as a calcium channel, it may involve the movement of endosomes containing Ebola virus or the membrane fusion.

Previous results had demonstrated that NPC1 mediates the entry of Ebola, and some small molecular inhibitors block the infection of Ebola by disturbing the interactions between NPC1 domain C and Ebola primed GP^9^. Thus, we first speculated that the CADs with selective anti-Ebola activity may interact with NPC1 to disturb the interaction between NPC1 domain C and Ebola primed GP. However, we did not detect any interactions of tamoxifen/clomiphene/U18666a with NPC1 domains A or C. In accordance with our results, another group also showed that clomiphene and U18666a do not disturb the interaction of NPC1 domain C and Ebola primed GP^3^. Recently, a study demonstrated that U18666a interacts with the NPC1 sterol-sensing domain (SSD) to inhibit NPC1 function and cholesterol transport; however, the authors emphasized that the inhibition of Ebola requires 100-fold higher concentrations of U18666A. They therefore excluded the possibility that U18666a interacts with the SSD of NPC1 to inhibit the Ebola entry^49^. Thus, these data demonstrate that NPC1 is not the target of CADs during Ebola entry inhibition.

Until now, approximately 80 FDA approved drugs have been shown to have selective anti-Ebola activities, and a structural analysis revealed that 45 of the 80 drugs are CADs (Supplementary Table S1). The majority of these CADs having selective anti-Ebola activity have the similar structural properties (pKa > 8, logP > 3) (Supplementary Fig. S6a). Based on their structural properties, most CADs are protonated, trapped, and finally concentrated in the acid endolysosome compartment^27^. Due to the liposolubility of CADs, they insert into the lipid bilayer of endolysosome and disturb the metabolism of lipids and the transport of several components, leading to the DIP, cholesterol accumulation, and/or functional inhibition of ASM (Supplementary Fig. S6b). Moreover, most CADs induce their incidental biological activities at micromolar concentrations, which match the concentrations of inhibition Ebola infection by CADs. Thus, it is possible that CADs inhibit Ebola entry through their incidental biological activities. A filipin staining and Ebola pseudovirion entry assay demonstrated that SERMs and U18666a induce the cholesterol accumulation and inhibit the Ebola entry at the same concentration (approximately 1 μM); however, the cholesterol accumulation (8 h) appeared after Ebola entry into the cytoplasm (2 h), implying that something changed within 2 h after the SERMs treatment to mediate the cholesterol accumulation and the inhibition of Ebola entry.

In contrast with this previous report, our data demonstrated that SERMs and U18666a decrease the sphingosine within 1 h. This discrepancy might be due to the different cell types and/or the different treatment dosages of U18666a used. In agreement with our results, much research has demonstrated that CADs can induce the detachment of ASM protein from the inner endolysosome membranes leading to inactivation. Several CADs have been identified as functional inhibitor of ASM^27^, and Elojeimy *et al*. have demonstrated that some CADs also inhibit the activity of AC^50^. Due to the metabolic pathways of sphingomyelin, the inhibition of ASM and AC both decrease the cellular sphingosine. It has been reported that Ebola infection requires ASM activity^51^, so CADs could inhibit Ebola entry through the inhibition of ASM activity. However, how ASM is involved in Ebola infection is still unknown; future studies are needed to better understand the molecular mechanism of ASM and Ebola entry.

It is well known that intracellular calcium signals involve in vesicular fusion, but whether the fusion of the Ebola and endolysosomal membranes also requires a calcium signal is still unclear. *In vitro*, the conformational change of membrane-bound Ebola fusion peptide, which is required for the vesicle fusion, is dependent on calcium concentrations^52^. Moreover, the proteins in endolysosomal calcium channels, TPC1 and TPC2, recently have been identified as key players in Ebola entry. The inhibitors for TPCs can block nicotinic acid adenine dinucleotide phosphate (NAADP)-stimulated calcium outflow and Ebola infection^47^. The sphingosine treatment could induce the deregulation of endolysosomal calcium^53^, and a subsequent study demonstrated that TPC1 and TPC2 (mainly TPC1) mediate the endolysosomal calcium response to sphingosine^54^. Consistent with these results, we proved that SERMs and U18666a decrease the sphingosine and increase the endolysosomal calcium. In addition, chelating luminal endocytic calcium with high-affinity Rhod-dextran also blocks Ebola infection, but not the internalization of Ebola VLP to TPC2+ lysosome. Taken together, these results demonstrated that the endolysosomal calcium is involved in Ebola infection and is also possible involved in the Ebola-endolysosome membrane fusion event.

Recently, Zhao *et al*. reported that toremifene can bind to and destabilize the Ebola GP trimer, triggering premature release of GP2, thereby preventing fusion between the viral and endolysosomal membranes^16^. This study revealed the mechanism of Ebola inhibition by toremifene, however, binding and destabilize GP may not play the major role in other situation. Firstly, the author informed that residues lining the binding site are highly conserved among filoviruses except Marburg virus (MARV), suggesting that MARV may not bind these drugs, but it has been proven that toremifene and clomiphene (even other CADs) could effectively inhibit the infection of MARV^4^,^6^. Secondly, compared to the ethyl chlorine of toremifene, the corresponding ethyl group in tamoxifen and chlorine in clomiphene make weaker interations with the binding sites in Ebola GP^16^, and the obvious structural differences of raloxifene (and other CADs with anti-Ebola activities) with toremifene make the interactions more weaker (Supplementary Fig. S1). However, all these drugs can effectively inhibit the Ebola infection^3^,^4^,^6^,^7^. Thirdly, many of CADs with anti-Ebola activities inhibit Ebola and/or MARV infection at similar kinetics (inhibition > 90% at 10 μM)^3^,^4^,^6^,^7^, which is hard explained by interaction with GP. Therefore, the binding with GP plays a major role in Ebola inhibition by toremifene, but may not in the Ebola or MARV inhibition by toremifene, tamoxifen, clomiphene, raloxifene or U18666a (even other CADs with anti-Ebola activities).

**Figure 6:**
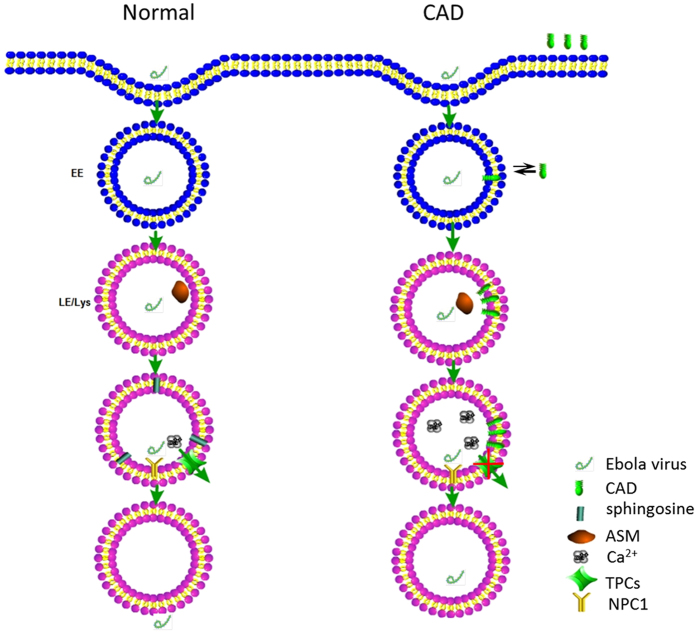
Proposed hypothesis of Ebola entry inhibition by CADs. Under normal conditions, Ebola is internalized by the NPC1+/TPC2+ endolysosome, in which the primed GP interacts with NPC1 domain C. The ASM and AC in the endolysosome then hydrolyze sphingomyelin to sphingosine. The elevated sphingosine induces endolysosomal calcium outflow through TPC1 and TPC2. Both the local calcium flux and the interaction of primed GP with NPC1 domain C induce conformational changes in the Ebola fusion peptide, triggering the membrane fusion during the Ebola infection. When treated with CADs that have selective anti-Ebola activities, including SERMs and U18666a, the CADs are protonated and trapped inside the acidic endolysosome. Due to their structural properties, these concentrated CADs induce the detachment of the ASM protein from membranes, which inactivates the ASM and in turn leads to a decrease in sphingosine. Without sufficient sphingosine, the endolysosome calcium outflow mediated by TPC1 and TPC2 are blocked, leading the endolysosome calcium accumulation. Without the calcium flux, there is no conformational change of the Ebola fusion peptide. Thus, there is neither membrane fusion nor Ebola infection.

In this study, we demonstrated that SERMs inhibit Ebola pseudovion entry and induce cholesterol accumulation at equal dosages, and they can reduce the cellular sphingosine and upregulate the endolysosomal calcium levels which is regulated by sphingosine through TPC1 and TPC2 54, moreover, chelation of endolysosomal calcium significantly and specifically inhibited the entry of Ebola. Based on previous studies and our results, we proposed a hypothesis of the Ebola infection (Fig. 6). Under normal conditions, Ebola is internalized by the NPC1+/TPC2+ endolysosome^55^, in which the primed GP interacts with NPC1 domain C^34^. The ASM and AC in the endolysosome then hydrolyze sphingomyelin to sphingosine^27^. The elevated sphingosine induces endolysosomal calcium outflow through TPC1 and TPC2^54^. Both the local calcium flux and the interaction of primed GP with NPC1 domain C induce conformational changes in the Ebola fusion peptide, triggering the membrane fusion during the Ebola infection^34^. When treated with CADs that have selective anti-Ebola activities, including SERMs and U18666a, the CADs are protonated and trapped inside the acidic endolysosome^27^. Due to their structural properties, these concentrated CADs induce the detachment of the ASM protein from membranes, which inactivates the ASM and in turn leads to a decrease in sphingosine^27^. Without sufficient sphingosine, the endolysosomal calcium outflow mediated by TPC1 and TPC2 are blocked, leading the endolysosomal calcium accumulation. Without the calcium flux, there is no conformational change of the Ebola fusion peptide. Thus, there is neither membrane fusion nor Ebola infection.

## Materials and Methods

### Reagents

17β-Estradiol (E_2_), ICI 182,780 (ICI; an ER antagonist), tamoxifen, clomiphene citrate, U18666A, and filipin were purchased from Sigma-Aldrich (Sigma, US). D-erythro-sphingosine (Sph) and C17-d-erythro-sphingosine (C17-Sph) were purchased from Avanti Polar Lipids (Alabastar, US). High-affinity (with the strong calcium buffering) Rhod-dextran was purchased from Thermo Fisher (Life technology, US). DMSO (Sigma, US) was used as solvent for tamoxifen and clomiphene citrate, PBS was used for U18666a and high-affinity Rhod-dextran, and methanol (Sigma, US) was used for Sph and C17-Sph.

### Cell and plasmids

The human hepatoma cell line HepG2, Hela cervical carcinoma cell line and the human embryonic kidney (HEK) 293 T cell line were obtained from the Cell Resource Center, Peking Union Medical College (licensed from ATCC). HepG2 was maintained in Eagle’s minimum essential medium (Gibco Invitrogen, US), Hela and 293 T was maintained in Dulbecco’s modified Eagle’s medium high glucose (DMEM-HG; Gibco Invitrogen, US) supplemented with 10% FBS (Gibco Invitrogen, US) and 100 U/ml penicillin (Sigma, US) and 100 μg/ml streptomycin (sigma, US). The cells were cultured at 37 °C in a humidified atmosphere of 5% CO_2_.

The Ebola (Gueckedou strain) GP, Ebola VP40, VSV GP, WSN HA/NA and pNL4-3-Luc-R^−^E^−^ vector have been generated as described previously^56^,^57^. We deleted the mucin (313–463aa) of Ebola GP by overlap-PCR to reduce the cytotoxicity of Ebola GP. The TPC2-EGFP and LAMP1-BFP expression vector were constructed by inserting TPC2 and LAMP1 to pEGFP-N1 and pBFP-N1 vectors.

### Expression and purifications of the A and C domains of NPC1

The NPC1 domains A and C were constructed, expressed and purified as previously described^34^,^35^. Briefly, Sf9 cells infected with NPC1 domain A (23–252, N70Q/N122Q/N185Q) baculovirus were used to infect Hi-5 cells. After incubation for 96 h at 27 °C, cells were pelleted by centrifugation, and the medium was concentrated by tangential flow filtration. The concentrated medium was applied to a Ni-NTA column. Fractions containing NPC1-A were pooled, concentrated. The concentrated NPC1-A was then applied to a Superdex 200 gel filtration column.

The cDNA encoding NPC1 domain C (374–620) was cloned into pET21a vector. The NPC1 domain C was expressed in *Escherichia coli* strain BL21 (DE3) as an inclusion body and then refolded *in vitro* using the method previously described^58^. The refolded NPC1-C was then concentrated and purified by gel filtration on a HiLoad 16/60 Superdex 75 PG column (GE Healthcare).

### Isothermal Titration Calorimetry

All ITC experiments were carried out at 25 °C on an iTC200 instrument (MicroCal, US). The sample cell contained NPC1 domain A or C (30 μM), and tamoxifen/clomiphene/U18666a (300 μM of each compound) was added in 20 injections with 2 μL of each. Data were processed using Origin software (MicroCal, US). Each experiment was duplicated three times.

### Microscale Thermophoresis

Affinity measurements using MST were carried out with a Monolith NT.115 instrument (NanoTemper Technologies, Germany). NPC1 domain A was labeled using the NanoTemper NHS NT-647 labeling kit (NanoTemper Technologies, Germany). The labeling reaction was performed according to the manufacturer’s instructions and applying a final concentration of 20 μM protein with a 3x molar excess of dye at RT for 30 min in the dark. Free dye was eliminated using the supplied dye-removal columns equilibrated with the NPC1 domain A storage buffer. Tamoxifen, clomiphene or U18666a was diluted in the NPC1 domain A storage buffer creating a dilution series of 12 1:2 dilutions (100 μM to 0.56 nM for tamoxifen and clomiphene; and 10 μM to 0.06 nM for U18666a). For the thermophoresis experiment, each ligand dilution was mixed 1:1 with labeled NPC1 domain A, incubated for 2 min at RT, before applying samples to Monolith NT Standard Treated Capillaries (NanoTemper Technologies, Germany). Thermophoresis was measured at RT with 20% LED power and 20%/40% MST IR-laser power. Data from at least three independently performed experiments were analyzed (NT Analysis software version 1.5.41, NanoTemper Technologies, Germany) using the signal from Thermophoresis+ T-Jump. Each experiment was duplicated three times.

### Ebola pseudovirion and VLP preparation

For Ebola entry and trafficking assays, Ebola pseudovirion and VLPs were prepared by transfecting HEK 293 T cells using polyethyle-nimine with four plasmids: codon-optimized Δmucin (313–463aa) Ebola (Gueckedou strain) GP and HIV based pNL4-3-Luc-R^−^E^−^ for Ebola pseudovirion. Ebola GP, Ebola VP40 and Ebola VP40 with mCherry fused to its N terminus for Ebola VLP. The plasmids were transfected at a ratio of 1:1 and 1:1:1, respectively. VSV and WSN pseudovirions were generated in an identical fashion, but with the substitution of a plasmid encoding VSV GP or WSN HA/NA. Culture supernatant at 24 hour posttransfection (hpt) was collected and replaced with the fresh media. A second harvest was conducted at 48 hpt. All harvests were pooled and cleared of cell debris by two sequential centrifugations at 1070 g for 10 min at 4 °C. VLPs in the cleared supernatant were then pelleted through 20% sucrose [in 20 mM Hepes, 130 mM NaCl, pH 7.4 (HNB)] at 112,398 g (25,000 rpm) in an SW28 rotor for 2 hours at 4 °C. Pelleted VLPs were then resuspended overnight in 10% sucrose (in HNB; 1/100 of original volume of culture medium collected), aliquoted, and stored at −80 °C.

### Ebola entry assay

Approximately 24 h before the experiment, 5 × 10^4^ HepG2 cells were plated in each well of a 96-well dish. Cells were then pretreated for 30 min with the indicated concentration of inhibitor or with DMSO vehicle in medium without FBS. These drug concentrations were maintained in all subsequent steps of the experiment until lysis. After the preincubation period, the cells were infected with 50 μL of Ebola pseudovirion for 48 h and then lysed for the luciferase assay according to the manufacturer’s instructions (Galen, China).

### Ebola trafficking assay

HepG2 cells were transfected with TPC2-EGFP and LAMP1-BFP plasmids using Lipofectamine 3000 (Invitrogen, US). At 24 hpt, HepG2 cells were pretreated with DMSO, 10 μM tamoxifen, clomiphene, or U18666a for 1 h. HepG2 cells were then incubated with Ebola VLP for 2 h, and then fixed. The cells were mounted and then imaged by the Nikon A1 Confocal Microscope System (Nikon, Japan).

### Filipin staining

Cholesterol accumulation was monitored by staining HepG2 cells with filipin. The day before an experiment, 50,000 cells were plated on glass coverslips in a 24-well plate. The next day, cells were treated with inhibitors at the indicated concentrations for 24 h. After fixation with 4% paraformaldehyde, cells were washed twice with PBS, incubated in 50 mg/mL filipin (Sigma, US) in PBS for 1 h, and washed 3 times with PBS, after which the coverslips were mounted and imaged on a Zeiss Axio Observer fluorescence microscope (Carl Zeiss, Germany). Representative images were inverted for clarity and are shown with uniform adjustments to brightness and contrast across all images.

### Sphingosine measurement

We performed sphingosine measurements using LC–MS/MS (Thermo, US) as previously described^59^. Briefly, cells were washed twice with ice-cold PBS, then cell pellets containing approximately 1∼10 × 10^6^ cells were harvested by scraping cells with ice-cold PBS and transferred to pre-chilled 1.5 mL new eppendorf tube on ice. The suspended cells were centrifuged at 1000 rpm for 5 min at 4 °C to remove PBS, then cell pellets were resuspended in 100 μL distilled water for further sample preparation. Cell pellets were stored at −80 °C until use. 25 μL of HepG2 cell suspension, 10 μL of internal standards (10 ng/mL for C17-Sph) and 65 μL of methanol were added into a 1.5 mL eppendorf tube. The mixture was vortex-mixed for 10 s followed by centrifuged at 12,000 rpm for 5 min, then supernatant was transferred to clean glass vials, and 10 μL of the supernatant was injected into the LC–MS/MS system for analysis. The autosampler was set at 4 °C.

### Endolysosomal calcium assay

The endolysosome calcium assay was carried out as previously described with some modifications^53^. HepG2 cells were pre-incubated with 10 μM tamoxifen, clomiphene or U18666a for 1 h, and loaded with 5 μM membrane-permeable calcium indicators Fluo8-AM (Abcam, US) for 5 mins at room temperature, then placed into calcium-free buffer for confocal live imaging, repeating two different ROIs for each treatment. The 1 μM thapsigargin (TG, Sigma, US) was loaded at 60 s for 30 s to release the endoplasmic reticulum calcium, and 200 μM Gly-Phe β-naphthylamide (GPN, Sigma, US) was loaded at 330 s for 60 s to release the acidic compartment (endolysosomal) calcium. The GPN induced calcium release was quantified with Image J software version 1.42 (Image processing and analysis in Java, NIH, Bethesda, MD; http://rsb.info.nih.gov/ij/download.html).

### Statistical analysis

The data were analyzed for statistical significance with the SPSS 12.0.1 Package (SPSS Inc., US). Data for all groups were first tested for normality with the Shapiro-Wilk test. If the group’s data were normally distributed, they were compared using a one-way analysis of variance. *P* values < 0.05 were regarded as statistically significant. All values are presented as the means ± SEM (standard error of mean). All of the graphs were generated with GraphPad Prism 5.0 (GraphPad Software Inc., US).

## Additional Information

**How to cite this article:** Fan, H. *et al*. Selective inhibition of Ebola entry with selective estrogen receptor modulators by disrupting the endolysosomal calcium. *Sci. Rep.* **7**, 41226; doi: 10.1038/srep41226 (2017).

**Publisher's note:** Springer Nature remains neutral with regard to jurisdictional claims in published maps and institutional affiliations.

## Supplementary Material

Supporting Material
