# Supplementary material for: Selective inhibition of Ebola entry with selective estrogen receptor modulators by disrupting the endolysosomal calcium
Source: Sci Rep. 2017 Jan 24;7:41226. doi: 10.1038/srep41226 (PMC5259750; doi:10.1038/srep41226)
Supplement: Supporting Material [file srep41226-s1.doc]

**Supporting material**

**Selective inhibition of Ebola entry with selective estrogen receptor modulators by disrupting the endolysosomal calcium**

Hanlu Fan^1#^, Xiaohong Du^1#^ , Jingyuan Zhang^1#^, Han Zheng ^1#^ , Xiaohui Lu^1^ , Qihui Wu^2^, Haifeng Li^1^, Han Wang^3^, Yi Shi^3^, George Gao^3^, Zhuan Zhou^2^, Dun-Xian Tan^4^, Xiangdong Li^1^*

^1^State Key Laboratory of AgroBiotechnology, Faculty of Biological Sciences, China Agricultural University, Beijing, 100193, China

^2^State Key Laboratory of Biomembrane and Membrane Biotechnology and Beijing Key Laboratory of Cardiometabolic Molecular Medicine, Institute of Molecular Medicine and PKU-IDG/McGovern Institute for Brain Research and Peking-Tsinghua Center for Life Sciences, Peking University, Beijing 100871, China.

^3^CAS Key Laboratory of Pathogenic Microbiology and Immunology, Institute of Microbiology, Chinese Academy of Sciences, Beijing 100101, China

^4^The University of Texas Health Science Center at San Antonio Department of Cellular and Structural Biology, San Antonio, TX 78229-3900, USA

^#^These authors contributed equally to this work

**Corresponding author & reprint requests**

Xiangdong Li, State Key Laboratory of Agrobiotechnology, Faculty of Biological Sciences, China Agricultural University, Beijing 100193, China. [xiangdongli@cau.edu.cn](mailto:xiangdongli@cau.edu.cn), 86-10-62734389 (Tel/Fax)

**
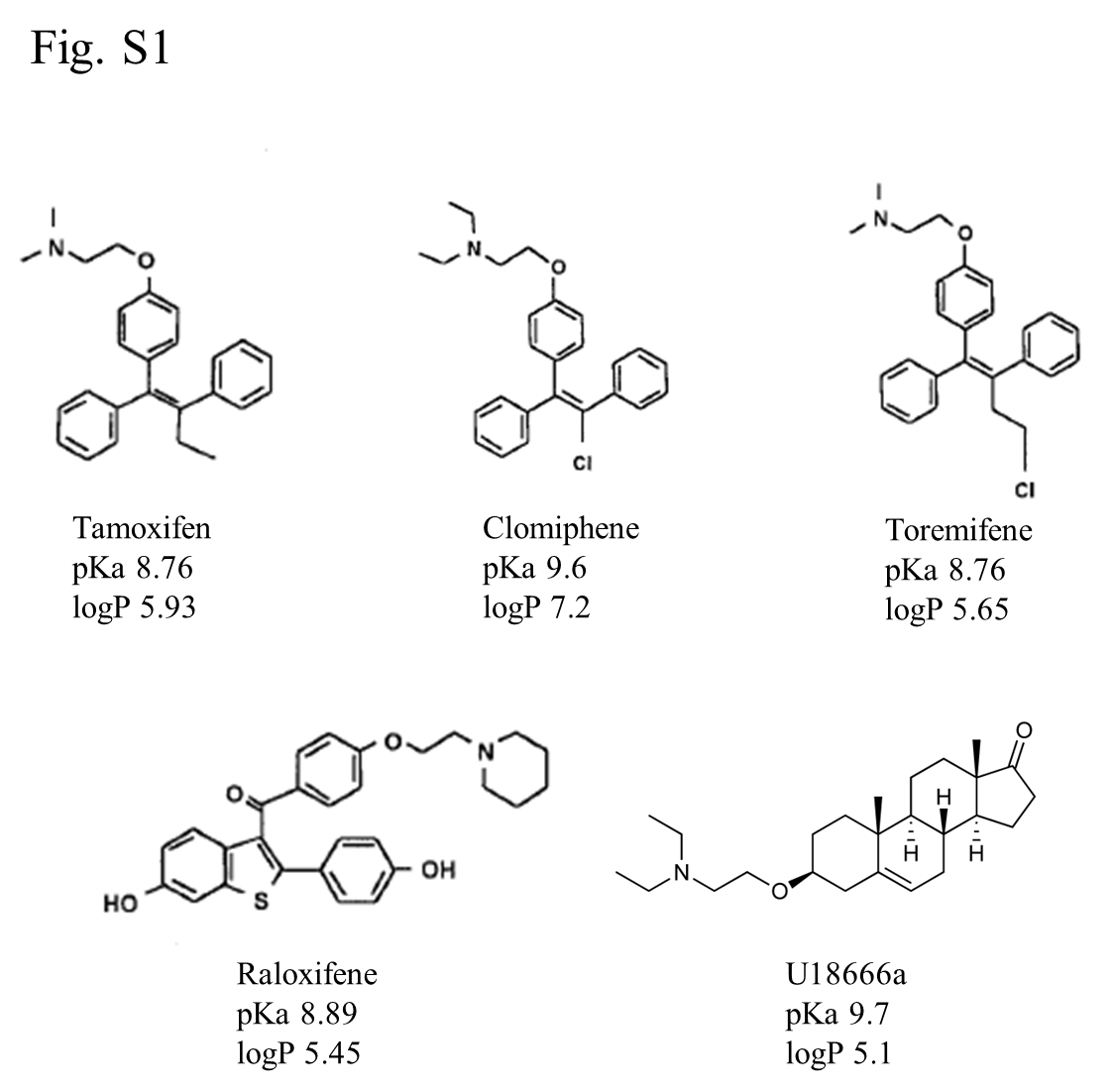
**

**Fig. S1** The structure of SERMs (tmaoxifen, clomiphene, toremifene and raloxifene) and U18666a.

Fig.S2


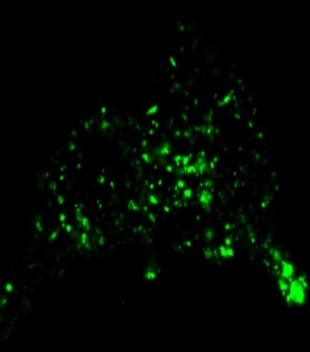

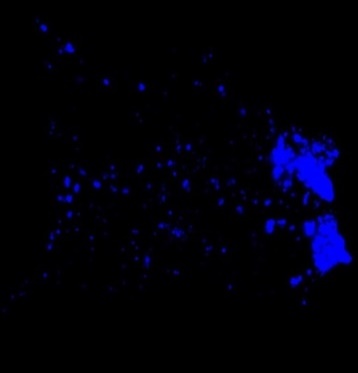

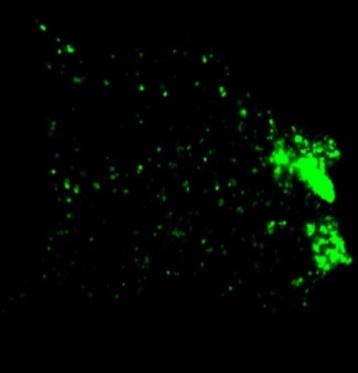

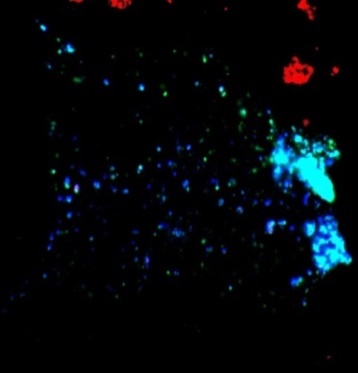

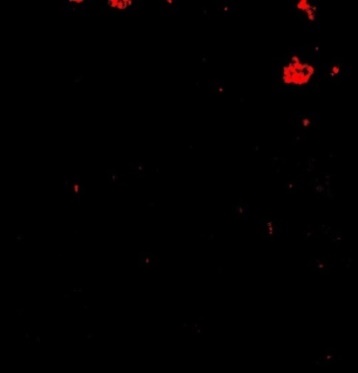

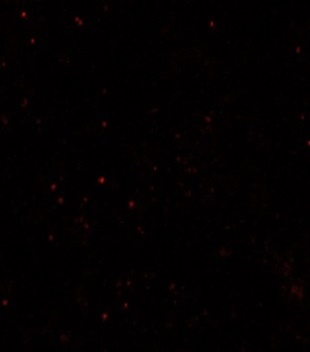

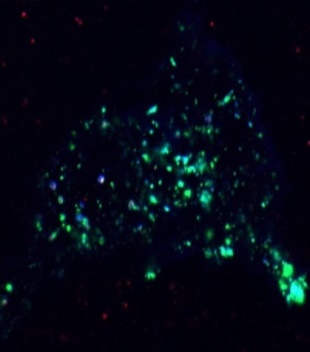

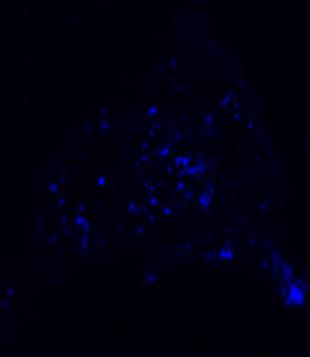


TPC2-EGFP LAMP1-BFP Ebola-VLP-mcherry Merge

U18666a Ctrl


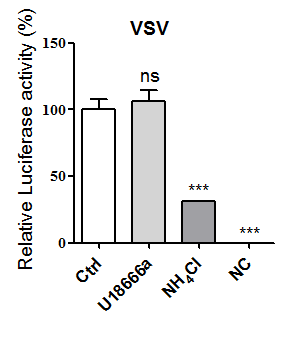

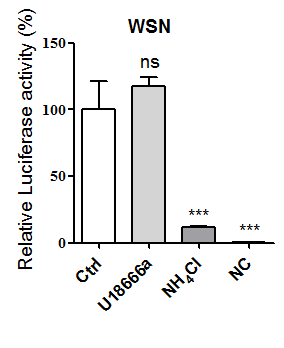

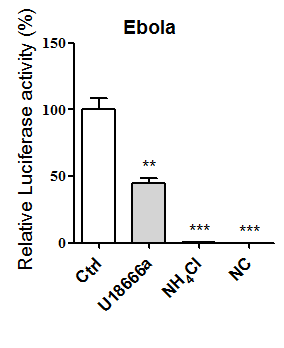


a

b

**Fig. S2** Effect of U18666a on Ebola/WSN/VSV pseudovirion entry and Ebola VLP internalization.

(**a**) HepG2 cells were pretreated with 10 μM U18666a for 1 h, and infected by Ebola pseudovirion with U18666a for 24 h, then was lysed and to carry out the luciferase assay. NH_4_Cl serves as a negative control. (**b**) Representative images of colocalization of VLPs (red, marked by mCheery-VP40) with TPC2 (green, marked with EGFP) and LAMP1 (blue, marked with BFP) from 10 μM U18666a treated HepG2. White arrows indicate examples of colocalization. Data are expressed as the means ± SEM (n = 3). Significant differences versus control group are presented by asterisks (*), ** *P* < 0.01, ns means no significant.

Fig.S3

a

b


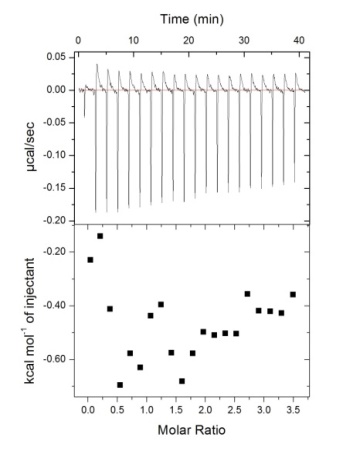

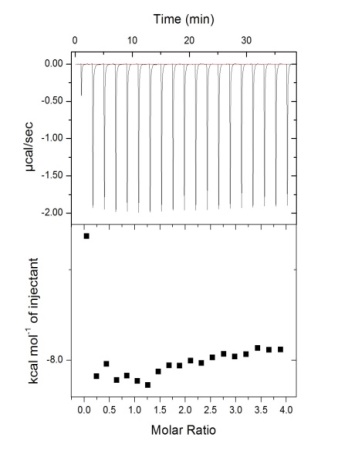

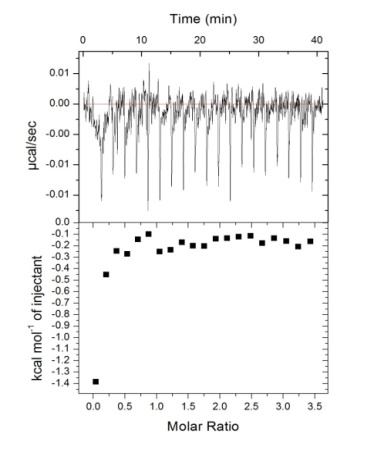

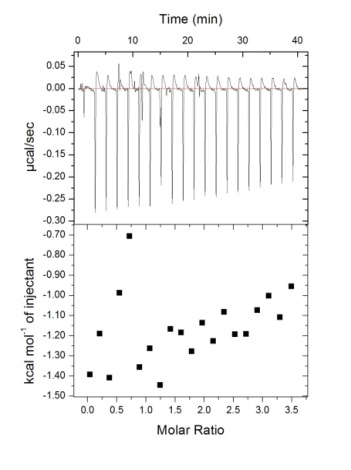

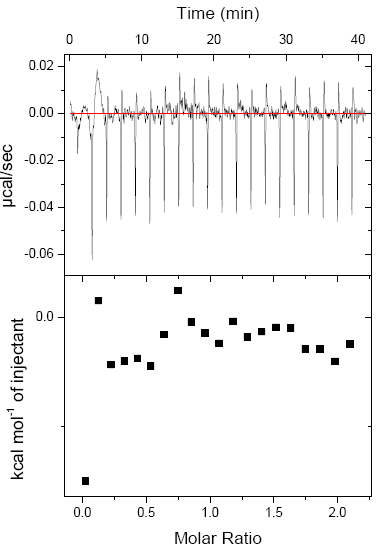

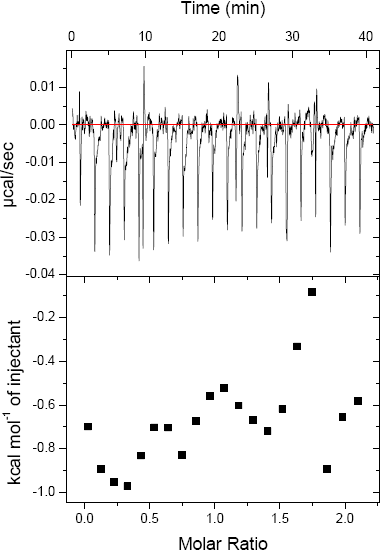


NPC1-

domain A

NPC1-

domain C

Tamoxifen Clomiphene U18666a


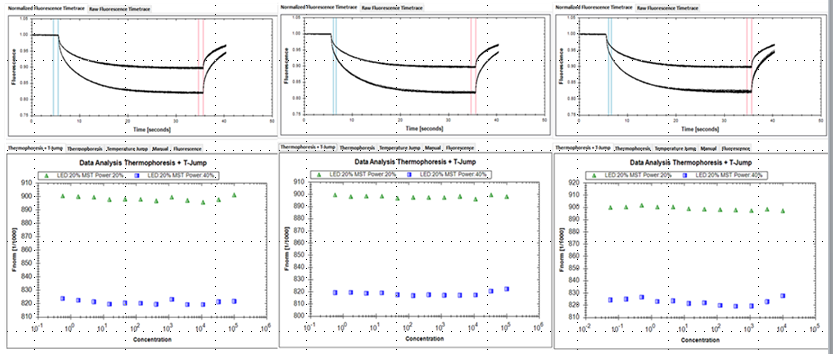


Tamoxifen Clomiphene U18666a

**Fig. S3** SERMs do not interact with NPC1 domain A or domain C.

(**a**) The ITC analysis of tamoxifen, clomiphene and U18666a binding to the NPC1 domains A and C. (**b**) The MST analysis of tamoxifen, clomiphene and U18666a binding to the NPC1 domain A.

Fig.S4


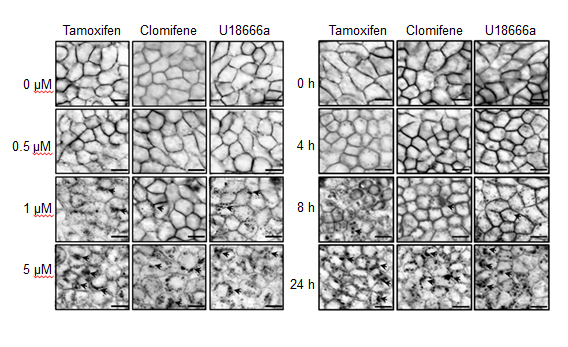


a

b

**Fig.S4** SERMs induce cholesterol accumulation in Hela cell.

(**a**) Dose-response cholesterol accumulation for tamoxifen, clomiphene and U18666a. (**b**) Time-response cholesterol accumulation for tamoxifen, clomiphene and U18666a. Black arrows indicate examples of cholesterol accumulation.

Fig.S5


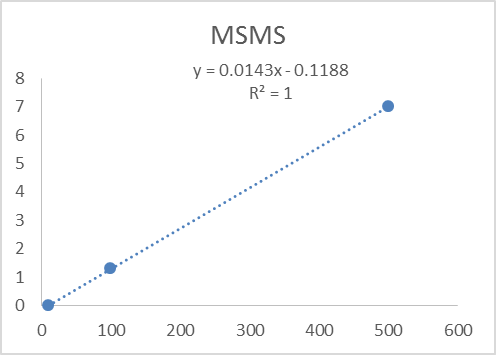

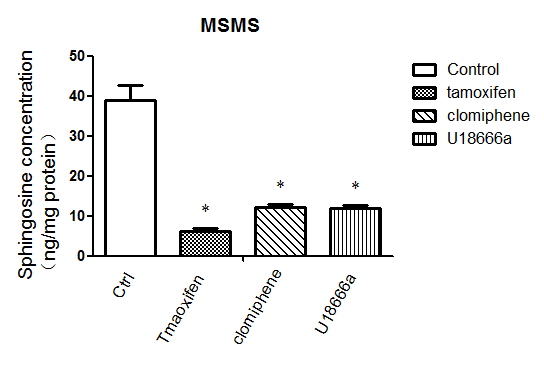


**Fig.S4** SERMs reduce the cellular sphingosine in Hela cell.

(**a**) Standard curves for quantification of Sph (0.1–100 ng/mL) with C17-Sph (10 ng/mL) as the internal standard. (**b)** Measurement of changes in intracellular levels of Sph in Hela cells. Cells were treated with 10 μM tamoxifen, clomiphene or U18666a for 1 h, then lipids were extracted and the levels of Sph were analyzed. Data are expressed as the means ± SEM (n = 3). Significant differences versus control group are presented by asterisks (*), * *P* < 0.05.


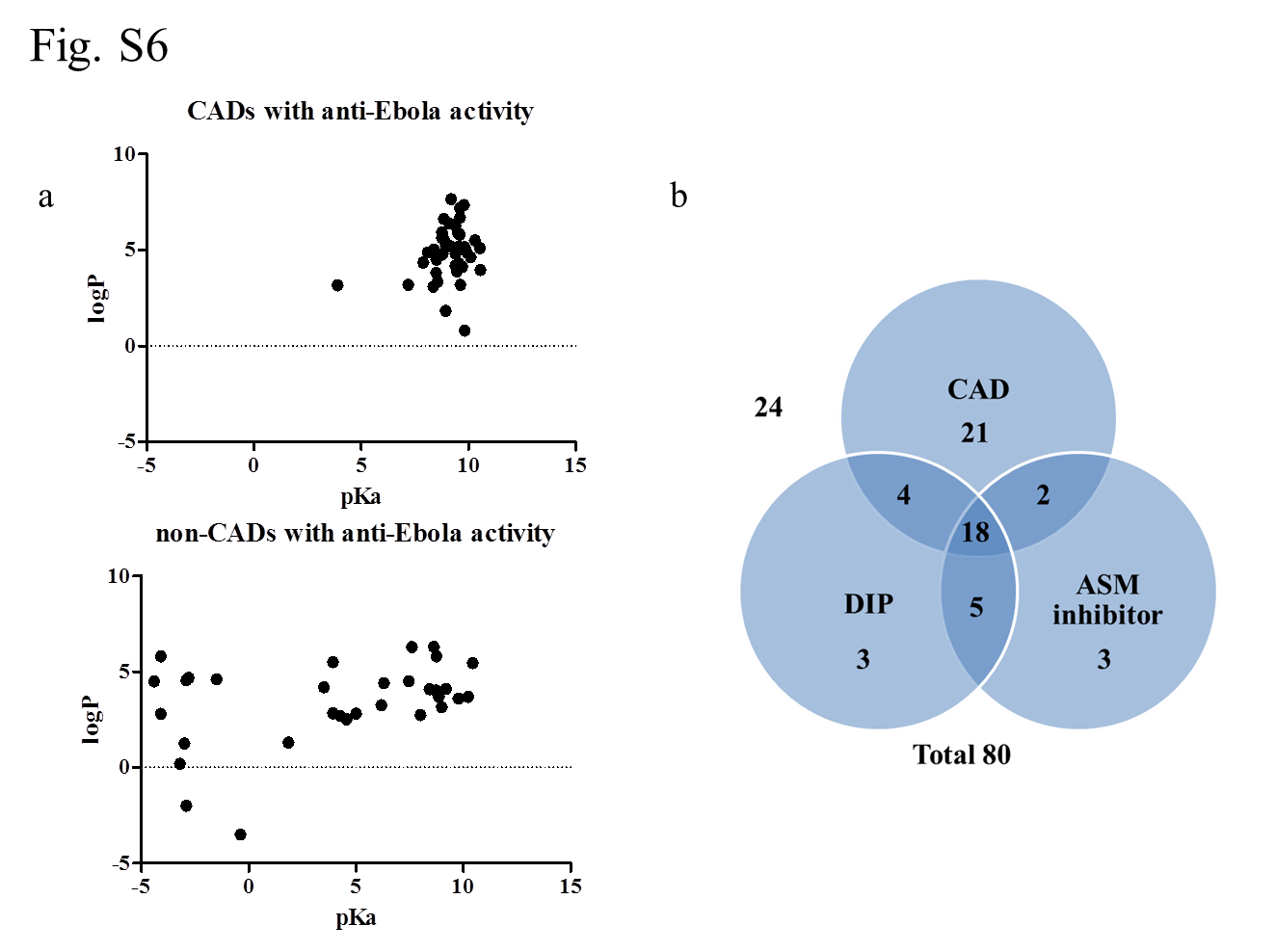


**Fig. S6** Physical properties and adverse effects of CADs with anti-Ebola activity.

(**a**) The structural analysis of the ex-FDA approved drugs with selective anti-Ebola activity. (**b**) The adverse effect of the ex-FDA approved drugs with selective anti-Ebola activity.

Table S1 The information of the ex-FDA approved drugs with anti-Ebola activity

| **compounds** | **pKa** | **LogP** | **cholesterol accumulation** | **DIP** | **ASM inhibitor** | **CAD** |
| --- | --- | --- | --- | --- | --- | --- |
| Albendazole | 4.27 | 2.7 |  |  |  | No |
| Alverine Citrate | 10.44 | 5.46 |  |  | Yes[^1^](#_ENREF_1) | No |
| Amodiaquine | 10.23 | 3.7 |  | Yes[^2^](#_ENREF_2) |  | No |
| Aripiprazole | 7.46 | 4.5 |  |  |  | No |
| Astemizole | 8.75 | 5.8 |  | Yes[^3^](#_ENREF_3) | Yes[^1^](#_ENREF_1) | No |
| Atovaquone | -4.1 | 5.8 |  |  |  | No |
| Azacitidine | -0.38 | -3.5 |  |  |  | No |
| Azithromycin | 9.57 | 4.02 | Yes[^4^](#_ENREF_4) | Yes[^5^](#_ENREF_5) | Yes[^6^](#_ENREF_6) | No |
| Bosutinib | 8.43 | 4.09 |  |  |  | No |
| Carfilzomib | 4.96 | 4.2 |  |  |  | No |
| Cepharanthine | 7.61 | 6.29 |  | yes[^3^](#_ENREF_3) | Yes[^7^](#_ENREF_7) | No |
| Deslanoside | -3.2 | 0.2 |  |  |  | No |
| Digoxin | -3 | 1.26 |  |  |  | No |
| Efavirenz | -1.5 | 4.6 |  |  |  | No |
| Lomerizine | 6.18 | 3.26 |  |  |  | No |
| Maduramicin | -2.9 | 4.56 |  |  |  | No |
| Mycophenolate | -4.1 | 2.8 |  |  |  | No |
| Niclosamide | -4.4 | 4.49 |  |  |  | No |
| Nilotinib | 6.3 | 4.41 |  |  |  | No |
| Nitrovin |  | 2.9 |  |  |  | No |
| Oxibendazole | 4.56 | 2.52 |  |  |  | No |
| Paroxetine | 9.77 | 3.6 | Yes[^8^](#_ENREF_8) | Yes[^3^](#_ENREF_3) | Yes[^1^](#_ENREF_1) | No |
| Pimozide | 8.63 | 6.3 | Yes[^9^](#_ENREF_9) | Yes[^3^](#_ENREF_3) | Yes[^1^](#_ENREF_1) | No |
| Piperacetazine | 9.2 | 4.1 |  |  |  | No |
| Proglumetacin |  |  |  |  |  | No |
| Simvastatin | -2.8 | 4.68 |  | Yes[^10^](#_ENREF_10) |  | No |
| Spiramycin | 8 | 2.745 |  |  | Yes[^6^](#_ENREF_6) | No |
| Strophanthin | -2.9 | -2 |  |  |  | No |
| Vinblastine | 8.86 | 3.7 |  |  | Yes[^11^](#_ENREF_11) | No |
| Vincristine | 8.66 | 2.82 |  |  |  | No |
| Vinorelbine | 8.72 | 4 |  |  |  | No |
| Clarithromycin | 8.38 | 3.16 |  |  |  | No |
| Colchicine | -0.038 | 1.3 |  |  |  | No |
| Mebendazole | 3.93 | 2.83 |  |  |  | No |
| Posaconazole | 3.93 | 5.5 |  | Yes[^12^](#_ENREF_12) |  | No |
| Aprindine | 9.94 | 4.86 |  |  | Yes[^7^](#_ENREF_7) | Yes |
| AY 9944 | 9.1 | 6.4 | Yes[^13^](#_ENREF_13) | Yes[^14^](#_ENREF_14) | Yes[^15^](#_ENREF_15) | Yes |
| Azaclorzine |  | 6.18 |  | yes[^16^](#_ENREF_16) |  | Yes |
| Bazedoxifene | 8.85 | 6.63 |  |  |  | Yes |
| Benztropine | 9.54 | 4.3 |  |  | Yes[^1^](#_ENREF_1) | Yes |
| Bepridil | 9.16 | 5.2 |  | Yes[^3^](#_ENREF_3) | Yes[^1^](#_ENREF_1) | Yes |
| Bifemelane | 10.56 | 3.97 |  |  |  | Yes |
| Bitolterol | 9.59 | 5.8 |  |  |  | Yes |
| Chloroquine | 10.1 | 4.63 | Yes[^17^](#_ENREF_17) | Yes[^18^](#_ENREF_18) | Yes[^7^](#_ENREF_7) | Yes |
| Clemastine | 9.55 | 5.2 |  | Yes[^3^](#_ENREF_3) | Yes[^7^](#_ENREF_7) | Yes |
| Clomiphene | 9.6 | 7.2 | Yes[^19^](#_ENREF_19) | Yes[^3^](#_ENREF_3) | Yes[^1^](#_ENREF_1) | Yes |
| Clomipramine | 9.2 | 5.19 |  | Yes[^20^](#_ENREF_20) | Yes[^21^](#_ENREF_21) | Yes |
| Cyclomethycaine | 9.4 | 6.25 |  |  |  | Yes |
| Dasatinib | 8.49 | 3.82 |  |  |  | Yes |
| Daunomycin | 8.94 | 1.83 |  | Yes[^11^](#_ENREF_11) | Yes[^11^](#_ENREF_11) | Yes |
| Dronedarone | 9.79 | 7.346 |  |  |  | Yes |
| Flupenthixol | 8.51 | 4.51 |  | Yes[^22^](#_ENREF_22) | Yes[^7^](#_ENREF_7) | Yes |
| Fluphenazine | 7.9 | 4.36 |  | Yes[^3^](#_ENREF_3) | Yes[^7^](#_ENREF_7) | Yes |
| Gefitinib | 6.85 | 3.2 |  |  |  | Yes |
| Hycanthone | 8.69 | 4.72 |  |  |  | Yes |
| Imipramine | 9.4 | 4.8 | Yes[^23^](#_ENREF_23) | Yes[^3^](#_ENREF_3) | Yes[^24^](#_ENREF_24) | Yes |
| Maprotiline | 10.54 | 5.1 |  | Yes[^16^](#_ENREF_16) | Yes[^1^](#_ENREF_1) | Yes |
| Mefloquine | 9.46 | 3.9 |  |  |  | Yes |
| Mibefradil | 9.82 | 5.16 |  | Yes[^3^](#_ENREF_3) | Yes[^1^](#_ENREF_1) | Yes |
| Nocodazole | 3.9 | 3.17 | Yes[^25^](#_ENREF_25) | Yes[^26^](#_ENREF_26) |  | Yes |
| Prochlorperazine | 8.1 | 4.88 |  |  |  | Yes |
| Propafenone | 9.63 | 3.2 |  |  |  | Yes |
| Quinacrine | 10.3 | 5.5 | Yes[^27^](#_ENREF_27) | Yes[^28^](#_ENREF_28) | Yes[^29^](#_ENREF_29) | Yes |
| raloxifene | 8.89 | 5.45 | Yes[^19^](#_ENREF_19) | Yes[^3^](#_ENREF_3) | Yes[^7^](#_ENREF_7) | Yes |
| Ro 48-8071 | 8.8 | 5.7 | Yes[^13^](#_ENREF_13) |  |  | Yes |
| Salmeterol | 9.4 | 4.2 |  |  |  | Yes |
| Sertraline | 9.85 | 5.1 | Yes[^30^](#_ENREF_30) | Yes[^31^](#_ENREF_31) | Yes[^1^](#_ENREF_1) | Yes |
| Sunitinib | 8.95 | 5.2 |  |  |  | Yes |
| tamoxifen | 8.76 | 5.93 | Yes[^19^](#_ENREF_19) | Yes[^3^](#_ENREF_3) | Yes[^1^](#_ENREF_1) | Yes |
| Teicoplanin | 9.2 | 7.66 |  |  |  | Yes |
| Terconazole | 8.8 | 4.8 | Yes[^13^](#_ENREF_13) |  |  | Yes |
| Thioproperazine | 8.36 | 3.09 |  |  |  | Yes |
| Thioridazine | 9.5 | 5.9 |  | Yes[^32^](#_ENREF_32) | Yes[^1^](#_ENREF_1) | Yes |
| Thiothixene | 8.56 | 3.36 |  |  |  | Yes |
| Tilorone | 9.72 | 4.13 |  | Yes[^33^](#_ENREF_33) |  | Yes |
| Topotecan | 9.83 | 0.8 |  |  |  | Yes |
| toremifene | 8.76 | 5.65 | Yes[^19^](#_ENREF_19) |  |  | Yes |
| Trifluoperazine | 8.39 | 5.03 |  | Yes[^3^](#_ENREF_3) | Yes[^1^](#_ENREF_1) | Yes |
| Triparanol | 9.6 | 6.7 | Yes[^13^](#_ENREF_13) | Yes[^34^](#_ENREF_34) |  | Yes |
| U18666A | 9.7 | 5.1 | Yes[^13^](#_ENREF_13) |  |  | Yes |

The pKa and logP of drugs are from www.drugbank.ca.

**References**

1 Kornhuber, J. *et al.* Identification of new functional inhibitors of acid sphingomyelinase using a structure-property-activity relation model. *Journal of medicinal chemistry* **51**, 219-237, doi:10.1021/jm070524a (2008).

2 Hirst, L. W., Sanborn, G., Green, W. R., Miller, N. R. & Heath, W. D. Amodiaquine ocular changes. *Archives of ophthalmology* **100**, 1300-1304 (1982).

3 Muehlbacher, M., Tripal, P., Roas, F. & Kornhuber, J. Identification of drugs inducing phospholipidosis by novel in vitro data. *ChemMedChem* **7**, 1925-1934, doi:10.1002/cmdc.201200306 (2012).

4 Van Bambeke, F. *et al.* Lysosomal alterations induced in cultured rat fibroblasts by long-term exposure to low concentrations of azithromycin. *The Journal of antimicrobial chemotherapy* **42**, 761-767 (1998).

5 Liu, Y., Kam, W. R., Ding, J. & Sullivan, D. A. One man's poison is another man's meat: Using azithromycin-induced phospholipidosis to promote ocular surface health. *Toxicology* **320**, 1-5, doi:10.1016/j.tox.2014.02.014 (2014).

6 Lewis, C. A. Enteroimmunology: A Guide to the Prevention and Treatment of Chronic Inflammatory Disease. (2014).

7 Kornhuber, J. *et al.* Identification of Novel Functional Inhibitors of Acid Sphingomyelinase. *Plos One* **6**, doi:ARTN e23852

8 Lara, N., Baker, G. B., Archer, S. L. & Le Melledo, J. M. Increased cholesterol levels during paroxetine administration in healthy men. *The Journal of clinical psychiatry* **64**, 1455-1459 (2003).

9 Rajkumar, K., Martinuk, S. D., Agu, G. O. & Murphy, B. D. In vitro binding and utilization of lipoproteins by luteal cells from ferrets treated with dopaminergic drugs during pseudopregnancy. *General and comparative endocrinology* **67**, 282-291 (1987).

10 Thompson, K. L. *et al.* Comparison of the diagnostic accuracy of di-22:6-bis(monoacylglycerol)phosphate and other urinary phospholipids for drug-induced phospholipidosis or tissue injury in the rat. *International journal of toxicology* **31**, 14-24, doi:10.1177/1091581811430167 (2012).

11 Jaffrezou, J. P. *et al.* Inhibition of Lysosomal Acid Sphingomyelinase by Agents Which Reverse Multidrug-Resistance. *Bba-Mol Cell Res* **1266**, 1-8, doi:Doi 10.1016/0167-4889(94)00219-5 (1995).

12 Cartwright, M. E. *et al.* Phospholipidosis in neurons caused by posaconazole, without evidence for functional neurologic effects. *Toxicol Pathol* **37**, 902-910, doi:10.1177/0192623309348521 (2009).

13 Shoemaker, C. J. *et al.* Multiple cationic amphiphiles induce a Niemann-Pick C phenotype and inhibit Ebola virus entry and infection. *PloS one* **8**, e56265, doi:10.1371/journal.pone.0056265 (2013).

14 Miyamoto, S., Matsumoto, A., Mori, I. & Horinouchi, A. Relationship between in vitro phospholipidosis assay using HepG2 cells and 2-week toxicity studies in rats. *Toxicol Mech Method* **19**, 477-485, doi:10.3109/15376510903322834 (2009).

15 Yoshida, Y. *et al.* Reduction of Acid Sphingomyelinase Activity in Human-Fibroblasts Induced by Ay-9944 and Other Cationic Amphiphilic Drugs. *J Biochem-Tokyo* **98**, 1669-1679 (1985).

16 Haranosono, Y., Nemoto, S., Kurata, M. & Sakaki, H. Establishment of an in silico phospholipidosis prediction method using descriptors related to molecular interactions causing phospholipid-compound complex formation. *The Journal of toxicological sciences* **41**, 321-328, doi:10.2131/jts.41.321 (2016).

17 Matsuzawa, Y. & Hostetler, K. Y. Studies on drug-induced lipidosis: subcellular localization of phospholipid and cholesterol in the liver of rats treated with chloroquine or 4,4'-bis (diethylaminoethoxy)alpha, beta-diethyldiphenylethane. *Journal of lipid research* **21**, 202-214 (1980).

18 Muller-Hocker, J., Schmid, H., Weiss, M., Dendorfer, U. & Braun, G. S. Chloroquine-induced phospholipidosis of the kidney mimicking Fabry's disease: case report and review of the literature. *Human pathology* **34**, 285-289, doi:10.1053/hupa.2003.36 (2003).

19 Shim, J. S. *et al.* Inhibition of angiogenesis by selective estrogen receptor modulators through blockade of cholesterol trafficking rather than estrogen receptor antagonism. *Cancer letters* **362**, 106-115, doi:10.1016/j.canlet.2015.03.022 (2015).

20 Sgaragli, G. P., Della Corte, L. & Gremigni, D. Chlorimipramine-induced phospholipidosis: biochemical and pharmacokinetic observations in the rat. *Pharmacological research communications* **15**, 231-246 (1983).

21 Kim, Y. & Sun, H. ASM-3 Acid Sphingomyelinase Functions as a Positive Regulator of the DAF-2/AGE-1 Signaling Pathway and Serves as a Novel Anti-Aging Target. *Plos One* **7**, doi:ARTN e45890

22 Shahane, S. A. *et al.* Detection of Phospholipidosis Induction: A Cell-Based Assay in High-Throughput and High-Content Format. *J Biomol Screen* **19**, 66-76, doi:10.1177/1087057113502851 (2014).

23 Rodriguez-Lafrasse, C. *et al.* Abnormal cholesterol metabolism in imipramine-treated fibroblast cultures. Similarities with Niemann-Pick type C disease. *Biochim Biophys Acta* **1043**, 123-128 (1990).

24 Liangpunsakul, S. *et al.* Imipramine Blocks Ethanol-Induced Asmase Activation, Ceramide Generation, and Pp2a Activation, and Ameliorates Hepatic Steatosis in Ethanol-Fed Mice. *J Invest Med* **60**, 726-727 (2012).

25 Lange, Y., Ye, J., Rigney, M. & Steck, T. L. Dynamics of lysosomal cholesterol in Niemann-Pick type C and normal human fibroblasts. *Journal of lipid research* **43**, 198-204 (2002).

26 Tyteca, D. *et al.* Azithromycin, a lysosomotropic antibiotic, impairs fluid-phase pinocytosis in cultured fibroblasts. *Eur J Cell Biol* **80**, 466-478, doi:Doi 10.1078/0171-9335-00180 (2001).

27 Appelqvist, H. *et al.* Sensitivity to Lysosome-Dependent Cell Death Is Directly Regulated by Lysosomal Cholesterol Content. *Plos One* **7**, doi:ARTN e50262

28 Marceau, F., Roy, C. & Bouthillier, J. Assessment of Cation Trapping by Cellular Acidic Compartments. *Method Enzymol* **534**, 119-131, doi:10.1016/B978-0-12-397926-1.00007-X (2014).

29 Farooqui, A. A. Lipid Mediators and Their Metabolism in the Brain. (2011).

30 Kesim, M. *et al.* The effects of sertraline on blood lipids, glucose, insulin and HBA1C levels: A prospective clinical trial on depressive patients. *J Res Med Sci* **16**, 1525-1531 (2011).

31 Rainey, M. M., Korostyshevsky, D., Lee, S. & Perlstein, E. O. The antidepressant sertraline targets intracellular vesiculogenic membranes in yeast. *Genetics* **185**, 1221-1233, doi:10.1534/genetics.110.117846 (2010).

32 Lullmann-Rauch, R. & Scheid, D. Intraalveolar foam cells associated with lipidosis-like alterations in lung and liver of rats treated with tricyclic psychotropic drugs. *Virchows Archiv. B, Cell pathology* **19**, 255-268 (1975).

33 Halstead, B. W. *et al.* A clinical flow cytometric biomarker strategy: validation of peripheral leukocyte phospholipidosis using Nile red. *J Appl Toxicol* **26**, 169-177, doi:10.1002/jat.1120 (2006).

34 Kacew, S. & Reasor, M. J. Newborn Response to Cationic Amphiphilic Drugs. *Federation proceedings* **44**, 2323-2327 (1985).
